# Effect of different starch acetates on the quality characteristics of frozen cooked noodles

**DOI:** 10.1002/fsn3.2692

**Published:** 2022-01-28

**Authors:** Kangyi Zhang, Di Zhao, Xiaojing Ma, Dongxu Guo, Xiaofeng Tong, Yun Zhang, Lingbo Qu

**Affiliations:** ^1^ Center of Agricultural Products Processing Henan Academy of Agricultural Sciences Zhengzhou China; ^2^ Henan Agricultural University Zhengzhou China; ^3^ Henan University of Technology Zhengzhou China; ^4^ School of Chemistry and Molecular Engineering Zhengzhou University Zhengzhou China

**Keywords:** frozen cooked noodle, physicochemical properties, starch acetate, textural property

## Abstract

The physicochemical properties of starch acetates with an equal degree of substitution prepared from pea, corn, and wheat starch and their effects on frozen cooked noodle (FCN) quality were investigated. The result showed that the three kinds of starch acetates had different effects on the quality of FCN due to their different blue values, freeze‐thaw stability, and crystalline morphology analyzed by XRD (*p* < .05). The FCN with the addition of 20% CAS exhibited slow deterioration of textural properties during holding for 30 min. The analysis of the changes in the content of free SH group and glutenin macropolymer (GMP) demonstrated that the addition of CAS promoted protein disulfide cross‐linking and decreased protein mobility during holding. Fourier transform infrared spectroscopy (FT‐IR) revealed that FCN with the addition of CAS had low decrement in α‐helix and β‐sheet during holding, indicating that starch acetates contributed to the maintenance of the gluten network structure.

## Graphical abstract

Starch acetates with an equal degree of substitution have different effects on the quality of frozen cooked noodles (FCNs); acetylated corn starch (CAS), acetylated wheat starch (WAS), and acetylated pea starch (PAS) were effective in reducing cooking loss and improving springiness of FCNs. Starch acetates can be used as a novel additive in FCNs to provide specific functionality and alleviate the deterioration of quality during holding.

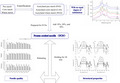

## 1 INTRODUCTION

Noodles originated in China and have been widely consumed for thousands of years. With the development of society and economy, consumers nowadays put forward higher requirements for ready‐to‐eat foods, such as convenience and health. As an emerging noodle product, frozen cooked noodles (FCNs) are widely accepted by consumers due to a short reheating time before eating. Meanwhile, with the development and application of chilling and quick‐freezing technology, the FCNs will show a great market potential.

The two main steps involved in the process of FCNs are precooking and freezing. Precooking involves boiling and steaming, which not only improved the springiness and chewability of boiled noodles but also solved the problems of the dry texture of steamed noodles (Luo et al., 2015; Shao et al., 2019), but did not improve the immersion resistance of noodles. Nowadays, FCNs are used more frequently for take‐out noodle products in China because of its convenience. However, cooked noodles lose chewiness within 30 min of delivery time, hampering its development in the take‐out market. Thus, the immersion resistance of noodles needs to be improved in order to avoid deterioration during the delivery time from fast food restaurants to consumers.

The quality of FCN was determined by multiple factors, such as the structure of the starch‐protein matrix, the moisture distribution, and starch gelatinization during cooking, freezing, and reheating (Liu et al., 2019; Wagner et al., 2011). The additive was helpful for the further development of FCN, which promoted the quality control. With low gelatinization temperature and good freeze‐thaw stability, modified starch can shorten cooking time and prevent ice crystal growth, thus helping to improve the color, texture, and tensile properties of noodles (Lin et al., 2019). Starch acetate is a starch derivative produced by the reaction of starch granules’ polyhydroxy structure with esterifying agents under the action of sodium hydroxide, pyridine, and other catalysts (Li et al., 2020). Compared with native starch, starch acetate has a wider range of functional properties such as increased peak viscosity (Li et al., 2020), paste transparency (Wani et al., 2012), and freeze‐thaw stability (Tang et al., 2018). There is a widespread use of starch acetate in the fields of frozen food, baked goods, sausage canned product, and so on (Xu et al., 2020; Zhang et al., 2014). The properties of acetylated starches depend on the botanical starch source (Chen et al., 2007). Olagunju et al. (2020) found that acetylation reduced the heat transition temperatures and retrogradation tendency, and significantly improved the physicochemical properties of acetylated pea starch (PAS), demonstrating its potential usefulness as a food additive. A previous study reported that acetylated corn starch could better meet the needs of the food industry by changing the properties of original starch, such as solubility, swelling power, water absorption, and transparency (Lin et al., 2017). Cao et al. (2021) found that the addition of acetylated cassava starch contributed to the formation of a more compact, uniform, and continuous comb‐like network. Additionally, Hong et al. (2020) modified wheat starch granules by dual esterification methods and found that acetylated wheat starch (WAS) has superior freeze‐thaw stability than native starch. Hu and Li (2021) confirmed this by adding 6%–9% chestnut starch acetate to dough, which improved the firmness and toughness of the frozen‐thawed dough.

In general, modified starch, as an improver, improved noodle quality. To our knowledge, few studies have been reported on the effect of starch acetate on FCNs. In this study, three different starches (pea, corn, and wheat starch) were used to prepare starch acetate with an equal degree of substitution (DS) and then applied to the preparation of FCNs. The objective of this study was to investigate different starch acetates in the texture of subsequently cooked noodles to determine whether it was possible to make a new type of convenient and nutritious FCNs.

## 2 MATERIALS AND METHODS

### 2.1 Materials

Starches were provided by Henan Hengrui Starch Technology Co. LTD. Wheat flour was purchased from Carrefour supermarket (Zhengzhou, Henan, China). Pancreatin, amyloglucosidase, and D‐Glucose Assay Kit were procured from Megazyme International Ltd. All chemicals and reagents used here were of analytical grade.

### 2.2 Starch modification

The starch sample (30 g) was dispersed in distilled water to make 30% (w/v) starch suspension, which was then adjusted to pH 8.5 with 3% (w/w) NaOH. Specific doses of acetic anhydride were added to the suspension to obtain modified starch with an equal DS. NaOH was used to maintain pH 8.5. Starch samples were placed in a microwave instrument with a cooling water circulating device (XH‐300UL, Beijing XiangHu Technology Development Co., Ltd) and treated with microwaves and ultrasonics simultaneously. The specific operation is as follows: the sample was under microwave and ultrasonic treatment for 5 s, and then just treated by ultrasonics for 55 s. The above process was performed by microwave at 500 w for 100 s and the ultrasonication with a frequency of 24.5 KHz at 500 w for 20 min. The slurry was cooled to room temperature. The obtained starch was allowed to dry in an oven at 45°C for 24 h. The wheat, pea, and corn starches under the coupled treatment by microwave and ultrasonication were named as WAS, PAS, and CAS, respectively.

### 2.3 Physicochemical characteristics of starch acetate

#### 2.3.1 Determination of degree of substitution

The DS of modified starch samples was determined using a titration method (Li et al., 2019). The native starch sample was used as a blank following the same process. The DS was calculated by the following equations:

(1) $$W\left(\%\right)=\frac{\left(V_{2}‐V_{1}\right)\times 10^{‐3}\times 43\times C}{m}\times 100$$

(2) $$DS=\frac{162\times W}{4300‐(43‐1)\times W}$$

where *W* is the mass fraction of acetic acid substituents (%), *V*_1_ is the volume of HCl required for the titration of the modified starch sample (ml), *V*_2_ is the volume of HCl required for the titration of the control sample (ml), *C* is the molarity of HCl solution (mol/L), and *m* is the quality of starch (g).

#### 2.3.2 Determination of blue value

The Iodine Binding procedure was based on that of Zhu et al. (2008). The absorption of the amylose–iodine complex was measured at 620 nm with a spectrophotometer.

#### 2.3.3 Determination of freeze‐thaw stability

Freeze‐thaw stability was determined by the method described by Yang et al. (2017). The syneresis rate was calculated as follows:

(3) $$\text{Syneresis}\;\left(\%\right)=\frac{\text{Weight}\;\text{of}\;\text{released}\;\text{water}}{\text{Weight}\;\text{of}\;\text{starch}\;\text{gel}}\times 100$$

#### 2.3.4 X‐ray diffraction analysis

Starch was subjected to X‐ray diffraction (XRD) analysis with an X‐ray diffractometer (D8 ADVANCE, BRUKER Corporation, Germany) using Cu‐Kα X‐rays at 1.54056 Å and an angle of diffraction scanning from 5° to 40° with a step length of 0.02°. The relative crystallinity was calculated as the proportion of the crystalline area to the total area multiplied by 100.

### 2.4 Preparation of frozen cooked noodles

The flour blends were prepared by mixing wheat flour and starch acetate. The flour blends were poured into a dough mixer container (HM740, Hauswirt), and an appropriate amount of water containing 1.5% sodium chloride was slowly injected. The mixture was stirred for 2 min at a slow speed and 10 min at a fast speed. The resulting dough was aged at 25°C and 80% relative humidity for 20 min. After resting, the dough was successively compressed several times at different roll gap settings, and the final sheet was cut into noodle strips about 1.0 mm thick and 2.0 mm wide using the noodle machine (JMTD 168/140, Beijing Dongfu Jiuheng Instrument Technology Co. LTD). Fresh noodles (30 g) were steamed in a steamer for 5 min and then boiled in 500 ml water for 3 min. The cooked noodle was cooled down by immersing it in 500 ml cold water (4°C) for 1 min, drained for 1 min, and put in a plastic bag for freezing in a −40°C refrigerator for 60 min. The frozen noodles were finally stored at −18°C for further analysis. Some fresh noodles and FCNs were freeze‐dried and ground into flour for subsequent analysis.

### 2.5 Cooking properties of FCNs

FCNs (20 g) were cooked in boiling water (400 ml) for 1 min. The water absorption of the noodles was determined as the ratio of the added weight of cooked noodles to total initial FCN weight. The collected gruel was transferred into a 500‐ml‐volume bottle, and the volume was fixed at room temperature. One hundred milliliters of the above gruel was precisely transferred into a beaker (predried to constant weight), and then the beaker was dried in an air oven at 105°C to constant weight. The weight of the residue is reported as the percentage of the noodle samples (calculated by dry basis).

### 2.6 Textural properties of FCNs

The textural properties of cooked noodles were measured using a TMS‐PRO texture analyzer (FTC, USA). Three cooked noodle strands were arranged adjacent to each other on the operating platform. The test conditions were as follows: texture profile analysis (TPA) mode; probe selection, P/50; parameter settings of pretest speed, test speed, and post‐test speed of TPA were 1.00, 1.00, and 1.00 mm/s, respectively; test compression rate of TPA, 75%; and trigger force, 5 g. Each sample was measured three times.

### 2.7 Determination of free sulfhydryl group content in FCNs

The sulfhydryl group contents of the samples were determined according to the method described by Rakita et al. (2014). The results were calculated using a standard L‐cysteine curve with dilutions between 0.02 and 0.08 μmol/ml.

### 2.8 Glutenin Macropolymer (GMP) in FCNs

The determination of GMP was conducted following a method reported by Li, Zhang, et al. (2019) with some modifications. Samples of 0.25 g were suspended in 5 ml 1.5% (w/v) SDS. The samples were rotated for 30 min at 30°C and then centrifuged at 15,500 g for 15 min at 15°C. The supernatant was decanted, and the remaining insoluble protein fraction was the GMP. The protein content of GMP and sample was estimated according to AACC Method 46‐10 (AACC, 2000) via the Kjeldahl apparatus (K9860, Hanon), which was used to calculate the protein content (N × 6.25). The ratio of the protein content in GMP to that in the corresponding sample is the GMP content.

### 2.9 Fourier transform infrared (FT‐IR) analysis

Fourier transform infrared spectroscopy (FT‐IR) of the sample was conducted, and the results were analyzed according to Jiang et al. (2014). The sample (approximately 1.5 mg) was mixed with 150 mg of KBr. FT‐IR spectra were recorded at wavenumbers of 4000 cm^−1^ to 400 cm^−1^ with a Thermo Nicolet Fourier transform infrared spectrometer (Nicolet iS5, ThermoFisher Scientific). The samples were subject to 64 scans per sample at a 4 cm^−1^ resolution. The secondary gluten structure was determined after Gaussian deconvolution and secondary derivation of the amide I region using Peak Fit v4.12 software. Secondary structure content was presented as a percentage of the corresponding area by ratio to the total amide I band area.

### 2.10 Statistical analysis

Mean values and standard errors of triplicate data were analyzed by ANOVA with SPSS 16.0. The multiple comparisons of data were determined by Ducan's test. Significance was defined at *p* < .05.

## 3 RESULTS AND DISCUSSION

### 3.1 Blue value

Amylose content is an important characteristic that greatly influences the functionality of starch (Mahmood et al., 2007). Apparent amylose of the starch samples was reflected by the blue value using iodine colorimetry. The blue value of PS was 0.733, much higher than that of CS and WS, which reflected that the amylose of the PS was the highest. The results were associated with the efficiency of the esterification reaction (shown in Table 1). The blue value represented the amylose content, which showed significant (*p* < .05) difference in native and treated starches as presented in Table 1. A significant decrease in blue value with esterification could probably be due to the fragmentation of amylose fraction, resulting in a reduction in the iodine‐binding ability (Shah et al., 2016).

**TABLE 1:** Dose of acetic acid anhydride, degree of substitution, blue value, freeze‐thaw stability, and relative crystallinity of native starches and their corresponding starch acetates

Sample	Dose of acetic anhydride (%)	DS	Blue value	Syneresis (%)			XRD
				1 cycle	3 cycles	5 cycles	RC (%)
PS	‐	‐	0.733 ± 0.004^a^	33.74 ± 5.33^a^	51.23 ± 5.69^a^	87.97 ± 4.11^a^	26.81
CS	‐	‐	0.656 ± 0.020^b^	26.15 ± 1.96^b^	44.91 ± 4.12^a^	79.61 ± 1.36^b^	24.00
WS	‐	‐	0.537 ± 0.012^c^	29.21 ± 2.18^ab^	48.72 ± 1.23^a^	85.95 ± 5.12^a^	22.42
PAS	5.5	0.080	0.472 ± 0.043^d^	16.22 ± 3.31^c^	35.72 ± 2.51^b^	42.30 ± 2.41^d^	22.32
CAS	4.8	0.081	0.245 ± 0.003^e^	8.12 ± 2.74^d^	24.21 ± 3.36^c^	36.80 ± 1.19^e^	22.18
WAS	5.0	0.081	0.459 ± 0.006^d^	13.14 ± 2.81^cd^	33.10 ± 4.18^b^	48.60 ± 1.96^c^	16.25

Note

Data are expressed as mean ± standard deviation.

Values in the same column with different letters are significantly different at *p* < .05.

Abbreviations: CAS, acetylated pea starch; CS, corn starch; PAS, acetylated pea starch; PS, pea starch; RC, relative crystallinity; WAS, acetylated wheat starch; WS, wheat starch.

### 3.2 Degree of substitution

The starch industry produces starch acetate on a commercial scale, characterized by a low DS with acetic acid residues, not exceeding 0.1 (Zi Eba et al., 2011). In this study, acetylated starch with an equal DS (ca. 0.08) was prepared from different types of native starch (pea starch, corn starch, and wheat starch). Different types of starches had different sensitivities to chemical modification, resulting in differences in the amounts of acetic acid anhydride used for esterification (Table 1). The amount of acetic acid anhydride reflected the efficiency of the esterification reaction. As can be seen from Table 1, the reaction efficiency of corn starch was the highest, mainly because the content of amylopectin in corn starch was higher, and the particles were larger than others, which makes the starch structure looser and easier to contact with chemical agents. Due to its high amylose content, pea starch molecules had a high degree of crystallinity (as shown in Table 1). It was difficult for chemical agents to enter the starch molecules, resulting in low reaction efficiency. Additionally, the various susceptibilities of amylose to chemical modification may indicate differences in the structure of starch crystallites formed during modification (Zięba et al., 2013). Therefore, during starch acetate denaturation, the efficiency of esterification will vary with different raw materials.

### 3.3 Freeze‐thaw stability

The freeze‐thaw stability, measured as syneresis, is regarded as an indicator of starch gels to assess their ability to resist undesirable physical changes during repeated freezing and thawing. And the freeze‐thaw stability was strong when the rate of water evolution was low (Hong et al., 2018). The freeze‐thaw stability of starch acetate is shown in Table 1.

It could be seen that CAS had a better freeze‐thaw stability and its syneresis rate was lower than PAS and WAS, which is consistent with previous research which reported that esterified starch granules with smaller amounts of amylose exhibited better freeze‐thaw stability (Hong et al., 2018). During repeated freeze‐thawing, the starch molecular chain can come close to each other, causing water molecules to be expelled (Zeng et al., 2019). Accordingly, branches of amylopectin starch can induce steric hindrance, which prevents water molecules from separating out of the gel network. The freeze‐thaw stability of starch gels was related to its retrogradation, and it was an important index for preparing starch‐rich frozen foods. We can conclude that CAS and PAS performed better than WAS under freeze‐thawing conditions. Accordingly, our findings provide a reference for syneresis potentials of these modified starches from the different botanical origins which would be applied to frozen foods.

### 3.4 X‐ray diffraction analysis

**FIGURE 1:**
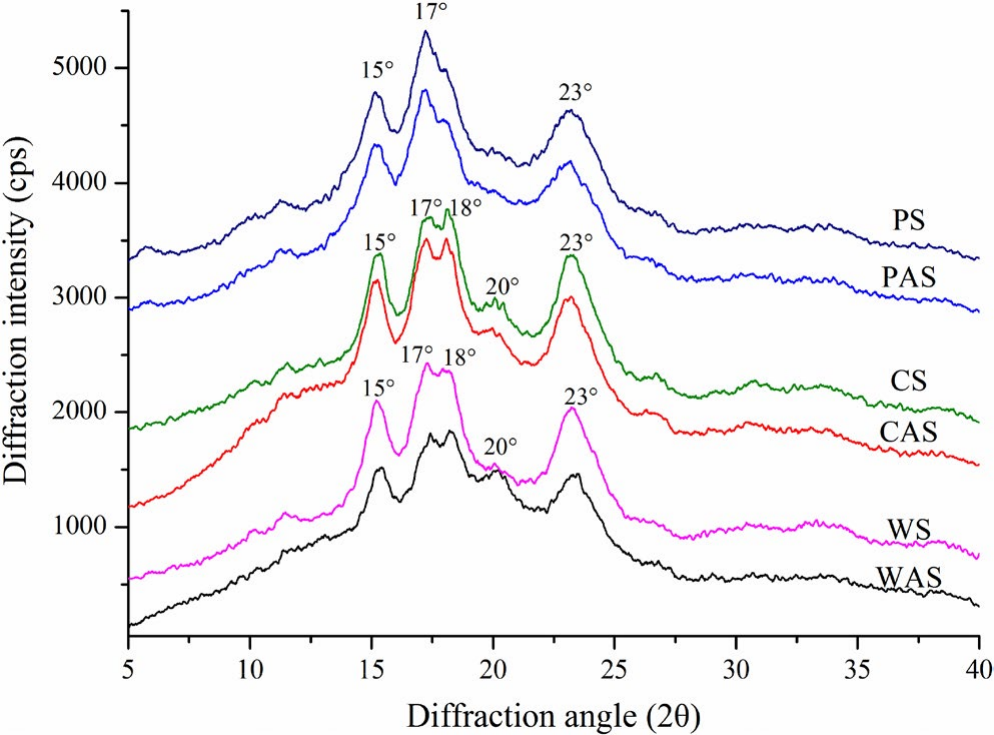
X‐ray diffraction patterns of native starches and their corresponding starch acetates. CAS, acetylated pea starch; CS, corn starch; PAS, acetylated pea starch; PS, pea starch; WAS, acetylated wheat starch; WS, wheat starch

X‐ray diffraction reflects the long‐range molecular order, generally termed crystallinity, which results from the ordered arrays of double helices formed by amylopectin side chains. The X‐ray diffraction patterns of native starch and starch acetate samples are presented in Figure 1 and the corresponding relative crystallinities (RC) are given in Table 1. Wheat starch and corn starch showed a typical A‐type crystalline structure, which exhibited main diffraction peaks at 2θ values of around 15°, 17°, 18°, 20°, and 23°, where the peaks at 17° and 18° exhibited a connected doublet. Meanwhile, PS exhibited a typical B‐type pattern, with strong reflections at 2θ about 15°, 17°, and 23°. After acetylation, the pattern was not changed. As shown in Table 1, RC of all the starch samples decreased after acetylation. This might be due to the introduction of new acetyl groups into the starch granule interior resulting in the damage of the ordered internal structure and depolymerization of amylopectin chain (Pietrzyk et al., 2018). The decrement in RC of wheat starch was higher than that of corn starch and pea starch, which indicated that it was easier for chemical agents to enter the starch molecules.

### 3.5 Cooking character of noodles

The cooking parameters, including best reheating time, cooking loss, and water absorption rate of the noodles, are shown in Table 2. A significant difference (*p* < .05) in the best reheating time was observed among FCNs with different starch acetates. The addition of three kinds of starch acetates shortened the reheating time of FCNs to varying degrees, and the reheating time gradually decreased with the increase in the amount of starch acetate. It was expected due to the increase in water absorption of noodles by adding the starch acetate, which reduced the energy of starch gelatinization and changed the best reheating time.

**TABLE 2:** Cooking character of the fresh noodles and FCNs with different starch acetates

Samples	Reheating time(s)	Cooking loss (%)		Water absorption (%)	
		Precooking	Reheating	Precooking	Reheating
Control	121.67 ± 4.04^a^	2.93 ± 0.03^e^	1.44 ± 0.10^a^	56.01 ± 0.22^ef^	83.91 ± ± 0.26^d^
PAS‐10%	89.67 ± 1.53^c^	3.03 ± 0.02^e^	1.06 ± 0.04^e^	61.72 ± 0.24^bcd^	93.94 ± 0.10^c^
PAS‐20%	74.67 ± 3.79^e^	3.22 ± 0.10^d^	1.11 ± 0.04^de^	59.31 ± 0.15^de^	94.82 ± 0.40^c^
PAS‐30%	66.67 ± 1.16^f^	3.36 ± 0.06^c^	1.20 ± 0.02^cd^	60.73 ± 0.61^cde^	95.63 ± 0.36^c^
CAS‐10%	102.67 ± 2.08^b^	3.35 ± 0.09^c^	1.11 ± 0.06^de^	66.54 ± 0.22^bcd^	94.21 ± 0.0^c^
CAS‐20%	83.00 ± 2.00^d^	3.24 ± 0.06^d^	1.18 ± 0.04^cd^	67.35 ± 0.28^bcd^	100.45 ± 0.36^bc^
CAS‐30%	73.67 ± 2.08^e^	3.13 ± 0.02^d^	1.02 ± 0.06^e^	69.51 ± 0.17^b^	106.85 ± 0.25^b^
WAS‐10%	103.33 ± 2.08^b^	3.45 ± 0.02^c^	1.22 ± 0.04^c^	62.81 ± 0.23^bcde^	100.55 ± 0.36^bc^
WAS‐20%	92.33 ± 1.53^c^	3.58 ± 0.09^b^	1.16 ± 0.03^cd^	68.42 ± 0.19^bc^	106.09 ± 0.29^b^
WAS‐30%	73.67 ± 1.16^e^	3.77 ± 0.12^a^	1.34 ± 0.06^b^	76.81 ± 1.26^a^	114.71 ± 1.20^a^

Note

Data are expressed as mean ± standard deviation.

Values in the same column with different letters are significantly different at *p* < .05.

The water absorption rate reflects the capacity of water absorbed during cooking. Noodles have an excellent texture only when the water absorption rate is appropriate. The water absorption rate of noodle samples increased with the addition of starch acetate, which might be because the added starch acetates filled the gluten network and enhanced its extensibility. Also, the addition of starch acetate formed certain hydrophilic groups, which improved the hydrophilicity of dough, thus promoting the crosslinking of starch molecules. Furthermore, prolonging the cooking time will lead to an increase in the boiled noodle weight and, therefore, an increase in the water absorption rate.

The cooking loss represents the number of particles diffused out from the samples into the noodle soup medium during boiling. A higher cooking loss indicates more dissolution in the noodle soup and worse cooking resistance (Shobha et al., 2015). With the addition of starch acetates, the loss rate of noodles was lower than that of control during reheating. However, the loss rate increased when the added amount reached 30%. This finding can be explained by the formation of a hydration network between starch molecules on the surface of starch particles, and stably adsorbing them into the noodle matrix (Han et al., 2014). The addition of excess starch acetates weakened the connection between gluten networks in noodles, which might lead to the dissolution of water‐soluble substances. Additionally, the cooking losses of reheated FCNs were much lower than that of the precooking noodles. It might be due to the short recooking time and the strong internal structure formed by starch gelatinization and protein denaturation in the early precooking process. The finding demonstrated that the short recooking time and the addition of acetates could reduce the cooking loss of noodles. The cooking quality of noodles was related to the cooking loss rate of noodles. The lower the loss rate, the better the quality (Lin et al., 2019). According to the requirements of the proper water absorption and low cooking loss, 20% starch acetate was considered the optimum additive amount for making FCNs after a comprehensive analysis.

### 3.6 Textural properties of noodles

**FIGURE 2:**
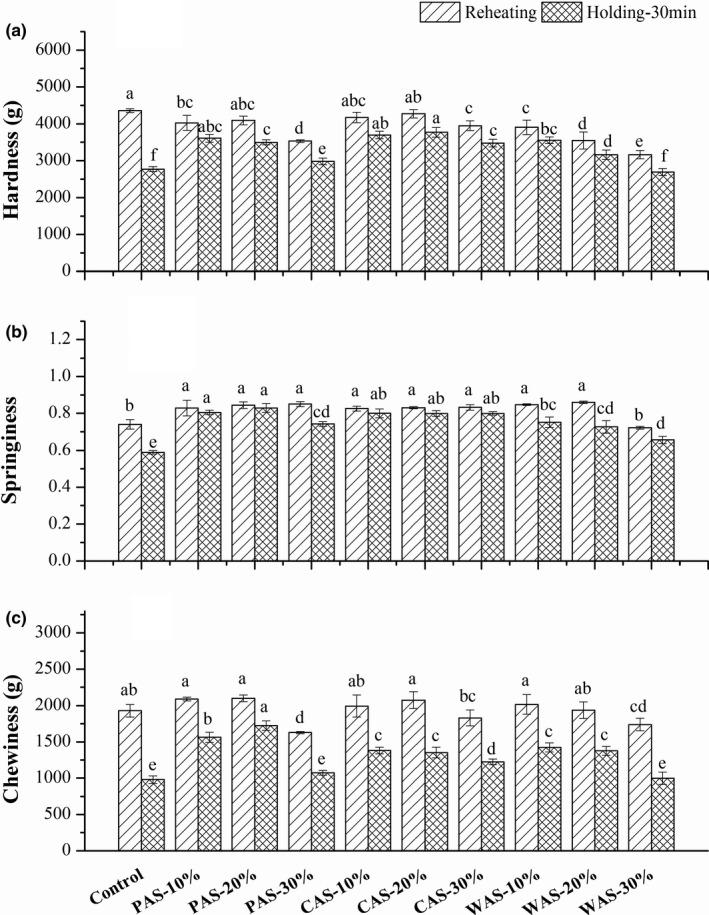
Textural properties of the reheated FCNs with different starch acetates after holding for 0 and 30 min. Different small letters in the same column indicate significant difference (*p* < .05)

Textural properties play a significant role in noodle quality, which affects final consumer acceptance. The effects of the three kinds of starch acetate on hardness, springiness, and chewiness of reheated noodles after holding for 0 and 30 min are summarized in Figure 2(a–c).

Compared with the control, a decrement was observed in the hardness of FCN with the addition of CAS, PAS, and WAS to some extent. The effect of starch acetate on textural properties was in agreement with that in Lin et al. (2017), who found a decrease in the hardness of the noodle during reheating. It was reported that the decrease in hardness was positively correlated with the content of gluten (Liu et al., 2018), and the addition of starch acetates led to the decrease in the gluten content of the noodle. Additionally, the high hardness of the control sample might be due to low water absorption during reheating, which led to the poor taste of noodles. Meanwhile, the hardness of noodles decreased with the increase of starch acetate. It might be related to the higher water absorption capacity of WAS, which promoted the swelling of starch granules, thus reducing the ability to withstand external pressure and significantly decreasing noodle hardness. During cooking, higher water absorption may lead to stickiness and poor elasticity in noodles, while lower water absorption may result in hardness and weak taste of noodles (Luo et al., 2015).

Chewiness reflects the energy required for chewing, which comprehensively reflects the continuous resistance of chewing noodle samples. As shown in Figure 2, the springiness and chewiness of reheated noodles decreased during holding. It might be due to the denaturation and agglomeration of protein during the cooking process of fresh noodles, leading to the reduction of gluten (Lin et al., 2019). Additionally, during the hot water soaking period, the degree of starch gelatinization will affect the water distribution, and the moisture in the outer high‐moisture area migrated to the inner low‐moisture area (Sekiyama et al., 2012). However, when the amount of starch added reached 30%, the quality of noodle products deteriorated. It might be due to the fact that the gluten network was not sufficient to cover the excess starch, leading to an increase in cooking loss and subsequent deterioration of quality. On the whole, during soaking in the hot soup, the addition of different starch acetates alleviated the decrease in hardness, springiness, and chewiness of FCN to some extent, indicating that a moderate addition of starch acetate slowed down the deterioration of FCN textural properties during holding. Considering the TPA results of FCN after holding for 0 and 30 min comprehensively, it was found that the addition of 20% starch acetate was most effective in improving the textural properties of FCN.

### 3.7 Free SH group

The changes in free SH content are an indicator of variation in SS bonds, which contribute to gluten aggregation (Wang et al., 2014). To further evaluate the formation of cross‐links between proteins during production and reheating of FCN, the levels of free SH groups in fresh noodles, FCN, FCN after reheating, and FCN after holding for 30 min were compared (Table 3).

**TABLE 3:** SH content, GMP, and protein secondary structure in the noodle

Samples		Free sulfhydryl (μmol/g)	GMP (%)	Protein secondary structure (%)			
				β‐sheet	α‐helix	β‐turn	(β‐sheet)+(α‐helix)
Control	Fresh noodle	1.645 ± 0.037^cd^	0.286 ± 0.000^e^	0.409	0.417	0.174	0.826
	FCN	1.549 ± 0.077^de^	0.521 ± 0.062^d^	0.386	0.390	0.225	0.775
	Reheating	1.468 ± 0.034^efg^	0.599 ± 0.015^a^	0.393	0.409	0.198	0.802
	Holding, 30min	1.511 ± 0.046^def^	0.561 ± 0.046^abcd^	0.382	0.391	0.227	0.773
PAS‐20%	Fresh noodle	1.821 ± 0.083^ab^	0.267 ± 0.004^e^	0.449	0.350	0.201	0.799
	FCN	1.340 ± 0.051^gh^	0.556 ± 0.016^abcd^	0.428	0.378	0.193	0.807
	Reheating	1.282 ± 0.016^h^	0.577 ± 0.012^abc^	0.463	0.366	0.171	0.829
	Holding, 30min	1.516 ± 0.139^def^	0.580 ± 0.012^ab^	0.450	0.369	0.181	0.819
CAS‐20%	Fresh noodle	1.754 ± 0.044^bc^	0.262 ± 0.001^e^	0.690	0.272	0.038	0.962
	FCN	1.240 ± 0.123^h^	0.532 ± 0.013^cd^	0.548	0.356	0.096	0.904
	Reheating	1.058 ± 0.015^i^	0.573 ± 0.005^abc^	0.466	0.354	0.180	0.820
	Holding, 30min	1.101 ± 0.010^i^	0.589 ± 0.002^ab^	0.449	0.372	0.185	0.815
WAS‐20%	Fresh noodle	1.950 ± 0.098^a^	0.219 ± 0.010^f^	0.525	0.329	0.145	0.855
	FCN	1.374 ± 0.008^fgh^	0.550 ± 0.036^bcd^	0.563	0.356	0.082	0.918
	Reheating	1.300 ± 0.177^h^	0.571 ± 0.010^abc^	0.477	0.356	0.167	0.833
	Holding, 30min	1.360 ± 0.140^gh^	0.581 ± 0.016^ab^	0.445	0.381	0.173	0.827

Note

Data are expressed as mean ± standard deviation.

Values in the same column with different letters are significantly different at *p* < .05.

For both noodle samples, a decrease in the amount of SH occurred in the process of producing FCN, and reduced again after reheating, while a slight increase was observed during holding for 30 min. It was probably due to the gluten aggregation during production through polymerization of the intrachain SH groups and the depolymerization effect of GMP via breakage of interchain SS bonds during the holding. This observation is in accordance with the results from GMP (Table 3). It indicated the cross‐linking of gluten through SS bonds occurred, and similar results were obtained by Lagrain et al. (2008), who also found a decrement in SH groups at high temperatures. Additionally, no significant differences were noted between free SH levels of FCN containing 20% CAS after reheating and those after holding, which indicated that the addition of CAS contributed to protein disulfide cross‐linking during holding.

### 3.8 Glutenin macropolymer (GMP)

The gluten protein consists of two major types of protein, monomeric gliadins and polymeric glutenins. Glutenins are the major components that confer strength and elasticity to the dough, while gliadins impart viscous properties (Wang et al., 2014). Among them, the insoluble glutenin with larger molecular weight was also called glutenin macropolymer (GMP), which has been demonstrated to play a prominent role in assessing dough properties and predicting noodle‐making quality.

As shown in Table 3, the GMP content in FCN samples and reheating noodles significantly increased (*p* < .05) above the value of fresh noodles. Although depolymerization of GMP was observed during the frozen storage in a previous study (Wang et al., 2014), the formation of large protein aggregates subjected to relatively high temperatures was found by Wang et al. (2016). It might be because that heat treatment promoted the conversion of free sulfhydryl groups into disulfide bonds, and the formation of disulfide bonds promoted the formation of polymer proteins. Additionally, the increase in GMP content indicated that part of gliadin was associated with glutenin through hydrophobic interactions during noodle cooking. The difference in GMP content reflected less depolymerization in fresh noodles, which was beneficial for maintaining better resilience to cope with starch swelling during cooking and reheating.

A slight increase in the GMP content in the reheating noodle during soaking was observed, except the control sample without starch acetate, owing to the decreased protein mobility (Table 3). Therefore, the mobility of the gluten network decreased because of the cross‐linking between glutenin and gliadin in reheating noodles. In addition, the gelatinization of embedded starch decreased the possibility of the isolation of the GMP. It can be related to the interplay between the impact of starch acetate on gluten network formation and at the same time on starch gelatinization.

### 3.9 Secondary structure of gluten

**FIGURE 3:**
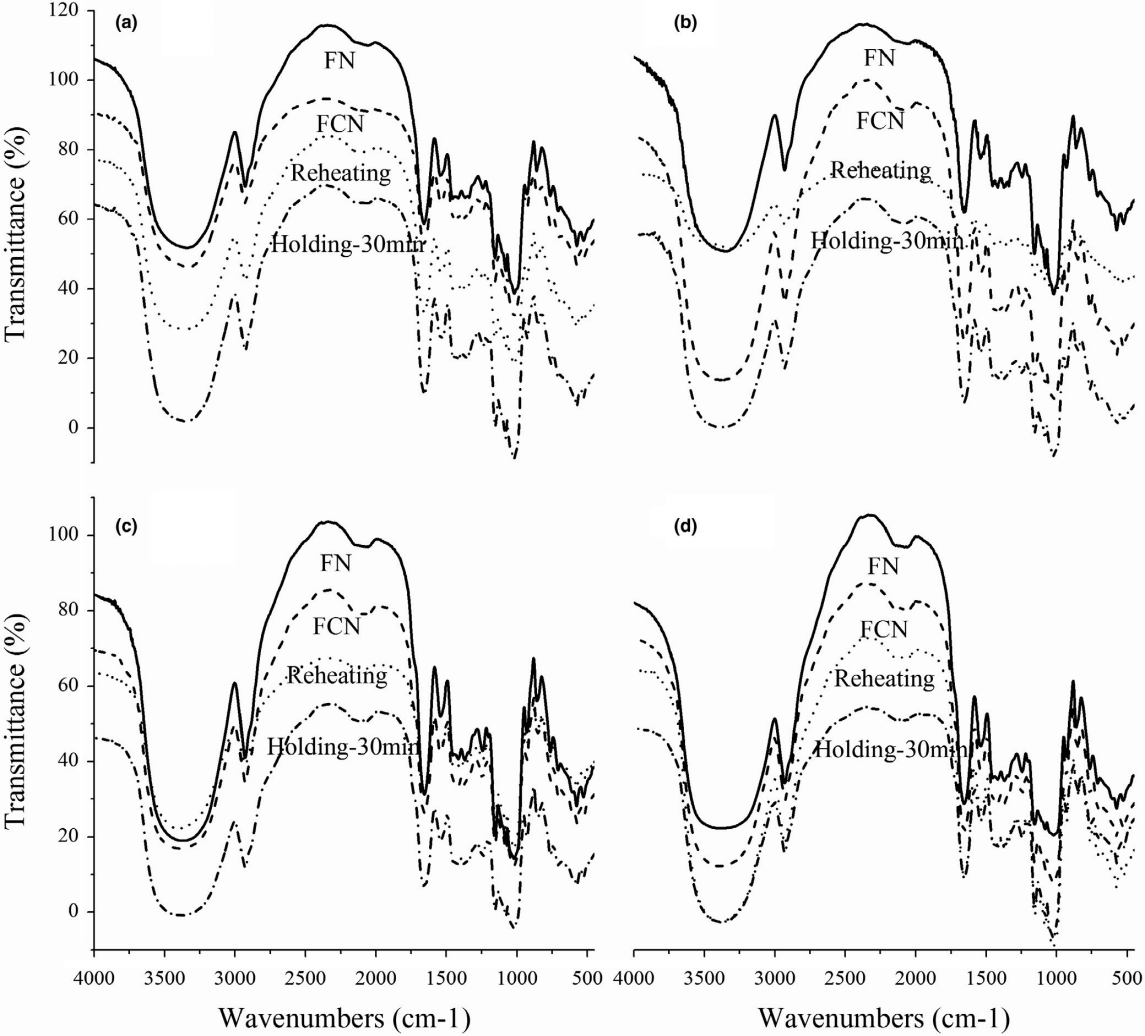
FT‐IR spectroscopy of the reheated FCNs with different starch acetates after holding for 0 and 30 min. a: Control FCN; b: FCN with the addition of 20% acetylated pea starch; c: FCN with the addition of 20% acetylated corn starch; d: FCN with the addition of 20% acetylated wheat starch

FT‐IR is widely used to evaluate the secondary structure of samples in many forms. Characteristic regions in the FT‐IR spectra of proteins mainly consist of amide I (1600–1700 cm^−1^) and amide II (1500–1600 cm^−1^). Amide I band is further investigated because the absorption associated with it is derived from stretching vibrations of the C = O bond of the amide, and it is a good predictor for quantitation of the protein secondary structure (Kumosinski & Farrell, 1993). The peak area corresponding to the different secondary structures was obtained (Figure 3), and the quantitative evaluation of secondary gluten structure changes in the noodle samples with different starch acetates during the process of frozen, reheating, and holding is presented in Table 3. No random coil structure was observed for all the samples.

The ratio of α‐helix and β‐sheet in the FCN sample with the addition of starch acetate was higher than that of control, which might be due to the better freezing thaw stability of starch itself after acetylation, causing less damage to the gluten network. A previous study found that the α‐helix structure was more hydrophobic, and increasing confirmation of α‐helix might lead to a more ordered structure. Thus, higher α‐helix content reflected greater water mobility and resulted in more dough stability (Li et al., 2014). Starch acetates induced changes in the protein secondary structure and thereby led to a more rigid protein network, causing the increase in hardness. Meanwhile, high β‐sheet content could enhance the intermolecular interactions by hydrogen bonds and promote protein aggregation, which was positively correlated with the viscoelasticity of dough (Bock et al., 2013). From Table 3, it can be observed that reheating the FCN samples increased the α‐helix fraction and decreased the fraction of β‐sheet and β‐turn contents significantly (*p* < .05), regardless of the type of starch acetate. This might be due to the conversion of less ordered β‐sheet and β‐turn structures into α‐helices, which possessed the compact organization during reheating. During holding, water migration interrupted the organized hydrogen bonding system, and thus both covalent and noncovalent bonds began to break, leading to depolymerization and the decrease in the ratio of α‐helix and β‐sheet. As shown in Table 3, α‐helix and β‐sheet in the control sample after reheating 30 min had a decrement of nearly 4%, while 1.2%, 0.6%, and 0.6% were observed in noodles with the addition of PAS, CAS, and WAS, respectively. It might be due to the high water absorption capability of starch acetate, which prevented the migration of water and maintained the secondary structure of proteins. In addition to changes in protein structure, molecular reorganization and degradation of starch granules were also crucial in the properties of gluten network structure (Turksoy et al., 2020).

Based on the analysis of the secondary structures, both CAS‐FCN revealed superior dough properties compared with the control and showed inferior dough characteristics based on the evaluation of noodle quality.

## 4 CONCLUSIONS

Starch acetates, with an equal DS, prepared from pea starch, corn starch, and wheat starch, had different properties, resulting in different effects on improving noodle quality. The addition of these three starch acetates effectively reduced cooking loss, enhanced water absorption, and further improved the textural properties of FCN. During holding, the increase in GMP indicated gluten protein aggregated, and the low decrement in α‐helix and β‐sheet showed the maintenance of the gluten network structure. The study indicated that starch acetate could be used as a modifying agent in the FCN process, especially for CAS.

## CONFLICT OF INTEREST

The authors have declared no conflict of interest.

## AUTHOR CONTRIBUTIONS

**Kangyi Zhang:** Conceptualization (supporting); Project administration (supporting). **Di Zhao:** Data curation (lead); Formal analysis (lead); Writing – original draft (lead); Writing – review & editing (lead). **Dongxu Guo:** Data curation (equal). **Xiaofeng Tong:** Formal analysis (equal). **Yun Zhang:** Formal analysis (supporting). **Lingbo Qu:** Supervision (supporting).

## Data Availability

The data used to support the findings of this study are available from the corresponding author upon request.
